# A Systematic Survey and Characterization of Enhancers that Regulate *Sox3* in Neuro-Sensory Development in Comparison with *Sox2* Enhancers

**DOI:** 10.3390/biology1030714

**Published:** 2012-11-22

**Authors:** Naoko Nishimura, Yoshifumi Kamimura, Yoshiko Ishida, Tatsuya Takemoto, Hisato Kondoh, Masanori Uchikawa

**Affiliations:** Graduate School of Frontier Biosciences, Osaka University, 1-3 Yamadaoka, Suita, Osaka 565-0871, Japan; Email: monster1116s@yahoo.co.jp (Y.K.); ishida-yoshiko@nst.hitachi-hitec.com (Y.I.); take@fbs.osaka-u.ac.jp (T.T.)

**Keywords:** *Sox3*, *Sox2*, enhancers, neuro-sensory development, functional assay, conserved sequence blocks

## Abstract

Development of neural and sensory primordia at the early stages of embryogenesis depends on the activity of two B1 Sox transcription factors, Sox2 and Sox3. The embryonic expression patterns of the *Sox2* and *Sox3* genes are similar, yet they show gene-unique features. We screened for enhancers of the 231-kb genomic region encompassing *Sox3* of chicken, and identified 13 new enhancers that showed activity in different domains of the neuro-sensory primordia. Combined with the three *Sox3*-proximal enhancers determined previously, at least 16 enhancers were involved in *Sox3* regulation. Starting from the NP1 enhancer, more enhancers with different specificities are activated in sequence, resulting in complex overlapping patterns of enhancer activities. NP1 was activated in the caudal lateral epiblast adjacent to the posterior growing end of neural plate, and by the combined action of Wnt and Fgf signaling, similar to the *Sox2* N1 enhancer involved in neural/mesodermal dichotomous cell lineage segregation. The *Sox3* D5 enhancer and *Sox2* N3 enhancer were also activated similarly in the diencephalon, optic vesicle and lens placode, suggesting analogies in their regulation. In general, however, the specificities of the enhancers were not identical between *Sox3* and *Sox2*, including the cases of the NP1 and D5 enhancers.

## 1. Introduction

Group B1 *Sox* genes, *Sox1*, *Sox2* and *Sox3*, encode HMG-domain transcription factors that show almost identical activity in the activation of target genes in transactivation assays [1,2,3], which is consistent with the model of their derivation from the same ancestral gene as a consequence of genome duplications [4]. In embryos, these B1 Sox factors regulate neuro-sensory development [5] in a functionally redundant manner when their expression overlaps in tissue. In most vertebrate species, the expression of *Sox2* and *Sox3*, which are activated in the early stages of embryogenesis, occurs prominently in the epiblast and neuro-sensory primordia [5,6,7,8,9,10,11,12,13]. In contrast, *Sox1* is activated at much later stages than *Sox2* and *Sox3* [5,6,7,14,15]. 

The most compelling evidence for the redundant functions and resulting functional compensation among B1 *Sox* genes has been provided by a study using zebrafish, where the developmental defects became evident only when activities of all B1 *Sox* genes expressed at early stages were downregulated [16]. In mouse embryos, the functional compensation between *Sox2* and *Sox3* has been indicated by the occurrence of embryonic lethality from *Sox2*+/-; *Sox3*-/- compound mutations, despite the viability of the *Sox2*+/- and *Sox3*-/- animals [17]. In addition, *Sox3* activity compensates for the loss of *Sox2* expression in the epiblast of post-gastrulation embryos [18].

The consequence of sharing of functions among the B1 *Sox* genes is that the targeted disruption of *Sox1*, *Sox2* or *Sox3* resulted in defective phenotypes only in the specific organs uniquely expressing these transcription factors. *Sox1*-mutant mice show abnormal lenses [19] and ventral striatum [20]. *Sox2*-mutant embryos die around the implantation stage, because *Sox2* is the only B1 *Sox* expressed at that stage [21]. *Sox3*-deficient embryos specifically develop abnormalities in the pituitary gland [22], gonad [23], and pharyngeal arch morphogenesis [17].

Functional dominance between *Sox2* and *Sox3* depends on the animal species. In amniotes, e.g., human, mouse and chicken, *Sox2* expression covers the entire neural primordia, and it is considered the lead transcription factor gene in primordial neural cells [24,25]. However, in lower vertebrates, *Sox3* expression is more prevalent in the neural primordia than *Sox2* expression [8,9,12,26], suggesting an evolutionary shift in the major regulatory processes in early neurogenesis from *Sox3*-centered regulation to *Sox2*-centered regulation in order to fulfill the equivalent B1 *Sox* functions [4]. Although the expression domains of *Sox2* and *Sox3* overlap extensively, their expression patterns are not identical, indicating differences in the regulation of these two B1 *Sox* genes. However, the regulation of *Sox2* and *Sox3* must be coordinated in the developmental processes that depend on the overall B1 Sox activity level.

Therefore, the regulation of the *Sox2* and *Sox3* genes is essential for the proper development of neural and sensory tissues. We previously identified more than 20 enhancers that are distributed in a 200-kb genomic region encompassing the *Sox2* gene [27,28]. Each of these *Sox2* enhancers was regulated in a unique manner, reflecting particular mechanisms that operate in specific tissues and/or at specific stages of neuro-sensory development [3,18,29,30]. Many of these enhancers are conserved in DNA sequences across vertebrate species [4,18,27,28], indicating strong conservation of the mechanisms of tissue regulation involving *Sox2* function. The N2 enhancer, which is responsible for *Sox2* activation in the epiblast and early anterior neural plate, is regulated by Zic, Pou and Otx factors [18,31]. In contrast, the *Sox2* N1 enhancer of *Sox2* is activated in the caudal lateral epiblast adjacent to the primitive streak. Axial stem cells, which are bipotential precursors for the neural plate and the paraxial mesoderm, reside in the caudal lateral epiblast [32], and produce neural and mesodermal cells, depending on whether *Sox2* or *Tbx6* is activated [33]. In addition, it has been shown that both axial stem cell maintenance and N1 enhancer activation depend on Wnt and Fgf signals [29,32,33,34]. 

In this study, we report a systematic survey and characterization of the enhancers that regulate the *Sox3* gene in neuro-sensory development. Many enhancers were found in the 231-kb span of the *Sox3*-encompassing chicken genomic region, as was the case for *Sox2* enhancers. In some cases, the enhancers were similarly regulated between the *Sox3* and *Sox2* genes. However, the varieties of enhancer specificities in spatial and temporal terms were substantially different between these genes. The *Sox3* NP1 enhancer that was activated in the caudal lateral epiblast was analyzed in detail because of its similarity to the *Sox2* N1 enhancer. The *Sox3* NP1 enhancer was found to be regulated by Wnt and Fgf signals, similar to the *Sox2* N1 enhancer, which is consistent with the essential involvement of Wnt and Fgf signaling in axial stem cell regulation [32]. 

A previous study using transgenic mouse embryos identified three putative enhancers within the genomic span, 3 kb upstream and downstream of the *Sox3* gene, which directs gene expression in distinct domains of the neural primordia [35]. In addition, an analogous *Sox3*-proximal DNA sequence of *Xenopus laevis* can reproduce a part of the *Sox3* expression patterns in transgenic frogs [12]. Our present study screened a wider genomic region and found more varieties of enhancers that regulate the *Sox3* gene in neuro-sensory development.

## 2. Results

### 2.1. Distribution of the Conserved Sequence Blocks (CSBs) in the Region Surrounding the Sox3 Gene Locus in Various Vertebrate Species

**Figure 1:**
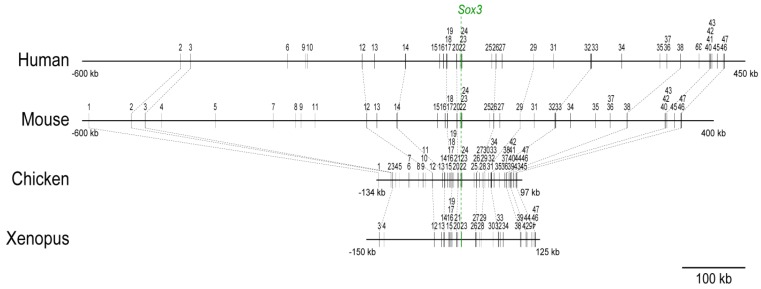
Comparison of the genome sequence organization of the *Sox3*-encompassing regions of human, mouse, chicken and Xenopus. The arrangement of the conserved sequence blocks (CSBs) that were numbered 1 to 47 in the region, between 134 kb upstream and 97 kb downstream, of the chicken *Sox3* gene. A CSB is defined as a sequence that shows >60% base identity compared with the chicken genome of more than a 100 bp sequence. The corresponding CSBs in different genomes bear the same numbers as in the chicken. The spacing between CSBs are similar for chicken and Xenopus genomes, but are widened in mammalian genomes. Human CSB1 positioned at -970 kb is not included in this Figure. Downstream, the correspondence between the mammalian and chicken sequences is lost beyond CSB47. The genomic coordinates of the CSBs in each genome are given in Table S1.

**Figure S1:**
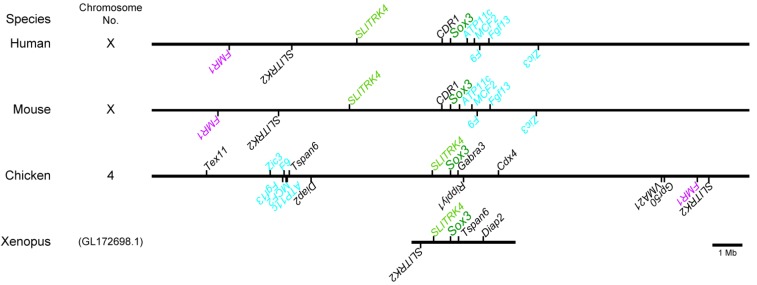
The arrangement of genes surrounding the *Sox3* locus in human, mouse, chicken and *Xenopus tropicalis*. The landmark genes are highlighted in different colors, indicating the occurrence of extensive rearrangements of the chromosomal segments among the animal species examined.

The *Sox3* gene is located on the X-chromosome in mammals, but it is autosomal in other vertebrate species. It is located on chromosome 4 in chicken, on scaffold GL172698.1 (without chromosomal assignment) in *Xenopus tropicalis*, on chromosome 14 in zebrafish, and on chromosome 10 in medaka (Figure S1). As the majority of enhancer sequences are included in the sequence blocks that are strongly conserved across the species (>60% DNA sequence identity over a length of 100 bp) [27], we compared the distribution of CSBs. As shown in Figure 1 and Table S1, many sequence blocks conserved between chicken and mammals and/or between chicken and *Xenopus* were identified both upstream and downstream of the *Sox3* gene. In fish genomes, a fraction of the blocks were identified, but the degrees of sequence conservation were generally low (data not shown). CSBs present in the chicken genome were numbered from 1 to 47. These blocks were located in the region that was between 134 kb upstream and 97 kb downstream relative to the *Sox3* open reading frame (ORF) start site (231 kb total), whereas in mammalian genomes the same conserved sequences were distributed in wider regions (940 kb in the mouse and 1.4 Mb in the human). Upstream CSBs from block 1 to 11 tend to be shorter (average 163 bp) compared to the entire CSBs (average 309 bp, excluding ~1 kb block 23 that included *Sox3* ORF). However, downstream of block 47, no CSBs were found between chicken and other animal species. From these observations, the 231-kb span of the chicken genome encompassing the *Sox3* gene was subjected to an enhancer survey.

### 2.2. Screening for Enhancer Sequences and Their Characterization

**Figure 2:**
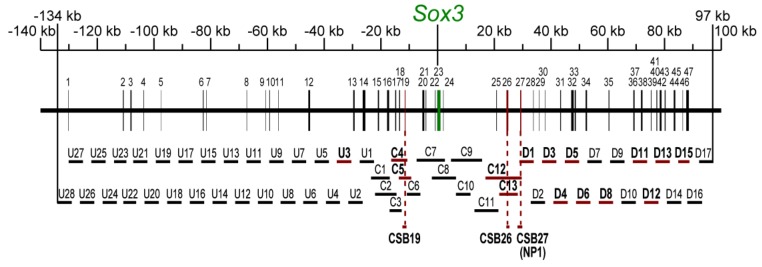
DNA sequences derived from the *Sox3*-encompassing genome region of the chicken and their analysis for enhancer activities. Distribution of CSBs 1-47 (black bars) upstream and downstream of the *Sox3* gene. CSB23 includes the *Sox3* open reading frame (ORF). The sequences of the central region, C1-C13, were derived from bacteriophage clones. The ~5 kb sequences that were upstream (U1 to U28) and downstream (D1 to D 17) with 1-kb terminal overlaps were derived from BAC clones. The DNA sequences indicated by red horizontal bars exhibited enhancer activity. CSBs 19, 26 and 27 that showed the same activity as the original DNA sequences are marked by red vertical bars. The nucleotide positions of these sequences and the statistics of the enhancer analysis are presented in Table S2.

**Figure 3:**
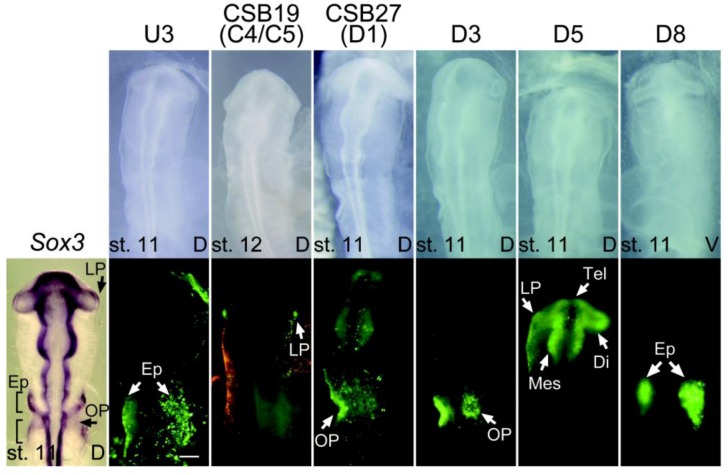
Enhancer activities in placode derivatives of the *Sox3*-flanking sequences or CSBs in electroporated chicken embryos at st. 11-12, as indicated by the EGFP expression, in comparison with *Sox3 in situ* hybridization (left). Top: Bright-field images with indication of developmental stages. V, ventral view; D, dorsal view. Bottom: EGFP fluorescence images of the same field. The bars indicate 200 μm. Di, diencephalon; Ep, epibranchial placode; LP, lens placode; Mes, mesencephalon; OP, otic placode; Rho, rhombencephalon; Tel, telencephalon.

**Figure 4:**
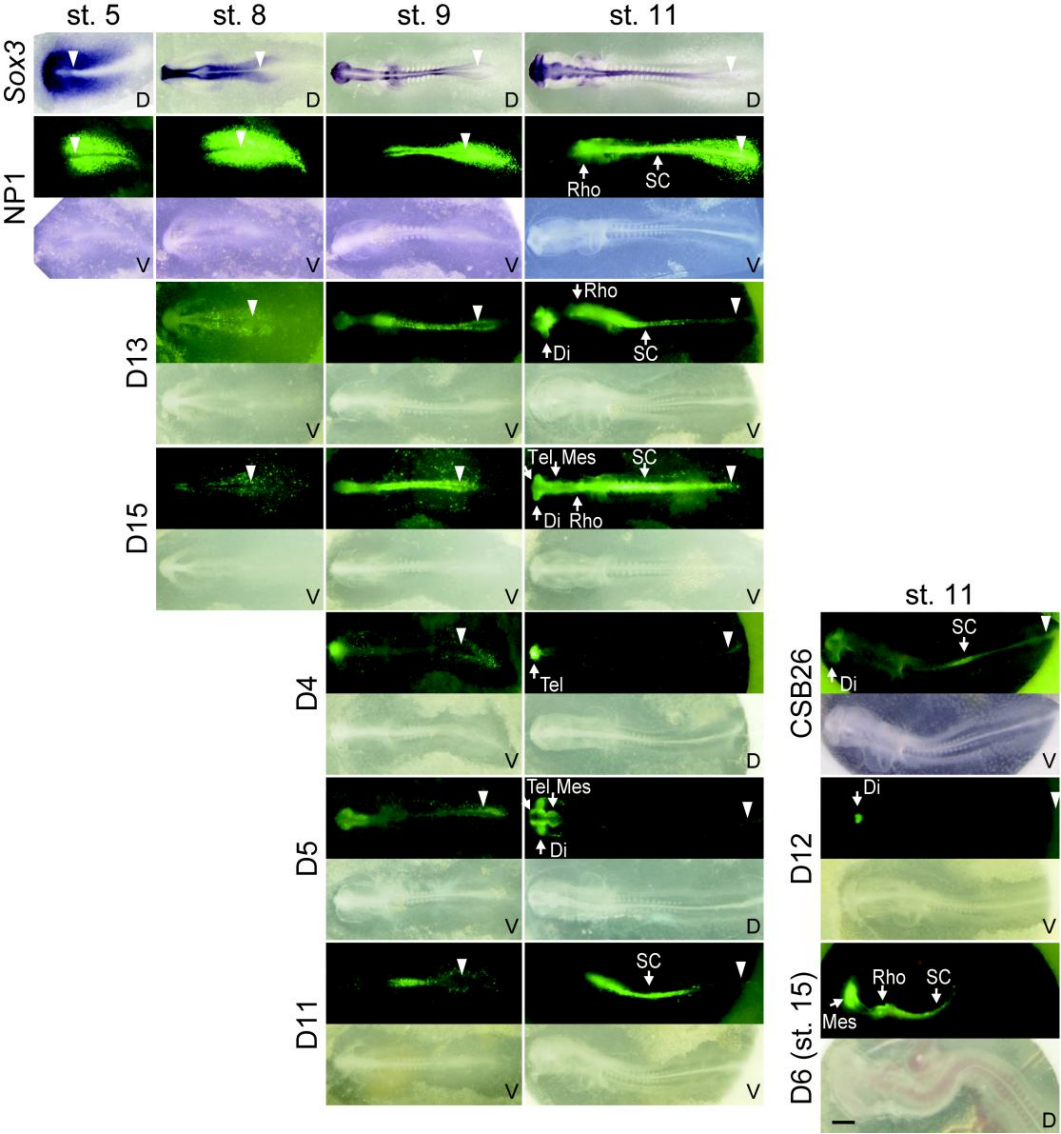
Sequential activation of *Sox3* enhancers that show distinct tissue specificities in the neural primordia. The D1 activity was represented by the NP1 enhancer sequence (see also Figure 5C). The top row: *Sox3 in situ* hybridization patterns in chicken embryos at respective developmental stages. Other rows, EGFP expression reflecting the specificity of the enhancers in comparison with the bright-field image of the same embryo. The data of an enhancer at different developmental stages was taken from the same embryo. V, ventral view; D, dorsal view. The white triangles indicate the positions of Hensen’s node. All photographs are shown on the same scale. The bar indicates 500 μm. Di, diencephalon; Mes, mesencephalon; Rho, rhombencephalon; SC, spinal cord; Tel, telencephalon.

We utilized the tkEGFP vector which by itself does not express EGFP upon electroporation in chicken embryos but expresses EGFP in a tissue-specific manner when an enhancer-bearing genomic fragment is inserted [27,36]. We initially analyzed the *Sox3*-proximal 50 kb region using bacteriophage clones derived from a chicken genome library. The overlapping DNA fragments C1 to C13 were prepared (Figure 2), inserted into the tkEGFP vector, and assessed for enhancer activities. Two regions of the genomic sequence exhibited enhancer activities: C4/5 in the st. 11 lens, and C12/13 in st. 11 diencephalon and spinal cord (Figure 3 and Figure 4, Table S2). The C12 sequence and the C13 sequence included in C12 exhibited identical enhancer activity. The C4/5 sequence included three CSBs (17, 18 and 19). The cloning of individual CSBs indicated that the CSB19 sequence was responsible for the lens enhancer activity of C4/5. The CSB26 sequence included in the C12 sequence exhibited enhancer activity in the diencephalon and spinal cord, which was identical to the activity of the C12 sequence.

These enhancers accounted for only subdomains of *Sox3* expression in embryos. Therefore, we extended the region of the enhancer survey to a 231-kb genomic span. We identified two BAC clones that covered the upstream and downstream regions of the central 50-kb region. We prepared a series of ~5 kb DNA sequences that spanned the upstream and downstream regions with 1 kb terminal overlaps, resulting in 28 upstream sequences (U1–U28) and 17 downstream sequences (D1–D17) that were external to the central 50-kb sequence (Figure 2), and examined their enhancer activities. The number of electroporated specimens for each DNA sequence is indicated in Table S2, where the same specificity of an enhancer was reproducibly demonstrated. 

**Figure 5:**
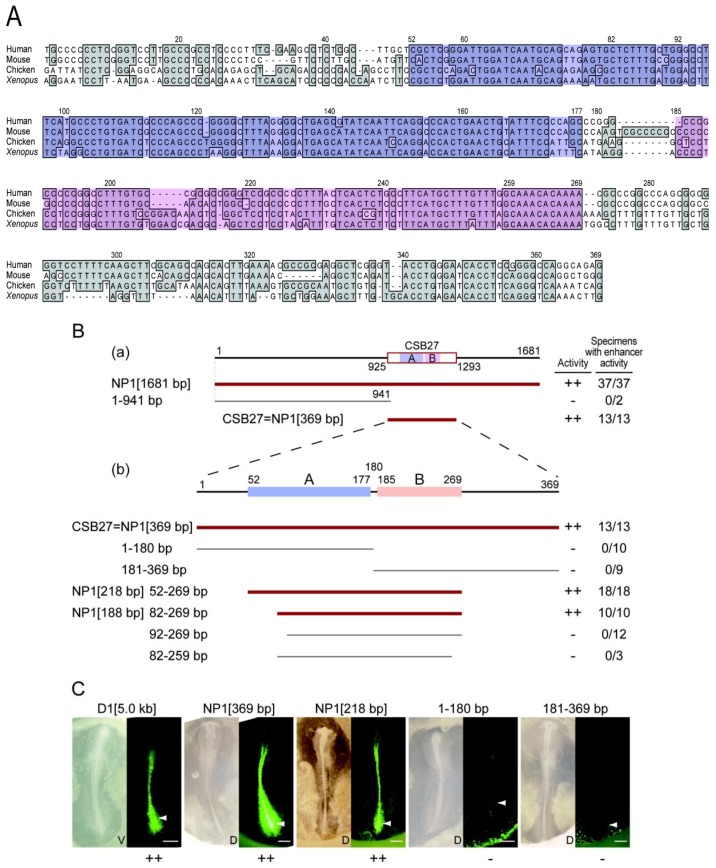
The sequence requirements for NP1 enhancer activity. **A.** Alignment of the CSB27 sequence of human, mouse, chicken and Xenopus. Two strongly conserved blocks, A and B, are highlighted in blue and pink, respectively. Other conserved bases are shaded in gray. The nucleotide positions in the chicken sequence are given at the top. **B.** Deletion analysis of the NP1 enhancer. The thick dark red lines indicate the DNA sequences showing full NP1 enhancer activity, while the thin gray lines indicate the sequences without enhancer activity. **(a)** A 1,681-bp DNA sequence (NP1[1681 bp]) that included the CSB27 sequence showed enhancer activity that was identical to the CSB27 (NP1[369 bp]) sequence. **(b)** The 5’ and 3’ halves of the CSB27 sequence, 1-180 bp and 181-369 bp, which included only one of the strongly conserved blocks, A and B, respectively, did not show NP1 enhancer activity. NP1[218 bp], consisting of the strongly conserved blocks A and B, displayed full NP1 activity. While a 30-bp deletion from the 5’ end (NP1[188 bp]) did not affect the enhancer activity, a 40-bp deletion (92–269 bp) inactivated the enhancer. The sequence of 82-259 bp with a 3’ 10-bp deletion from NP1[188 bp] lost enhancer activity. Thus, NP1[188 bp] was determined as the minimal DNA sequence that elicited NP1 enhancer activity that was definable from external deletions. **C.** Representative examples used to assess NP1 enhancer activity using chicken embryo electroporation. Both bright field and EGFP fluorescence images are shown for each sequence tested. D1 [5.0 kb], NP1[369 bp] and NP1[218 bp] sequences showed strong activity (++), while the 1-180 bp (including Block A) and 181-369 bp (including Block B) sequences were inactive (-). The bar indicates 500 μm.

In the upstream region, we identified only one sequence, U3, which showed enhancer activity in the epibranchial placode region at st. 11. However, in the downstream 97 kb region, ten sequences showed enhancer activities of various tissue specificities (Figure 3 and Figure 4, Table S2). The U3, D1, D3, D5 and D8 sequences showed enhancer activities in placode derivatives (Figure 3). The D3 sequence showed an otic placode-specific enhancer activity, while the D8 sequence displayed an epibranchial placode-specific activity that was similar to the aforementioned U3 enhancer. Other placodal enhancers also showed enhancer activity in the CNS as well.The D1, D4, D5, D6, D11, D12, D13 and D15 sequences showed enhancer activities in various portions of the developing CNS (Figure 4). The enhancer activity of the D1 sequence was of particular interest because of its strong activity in the caudal lateral epiblast, which is similar to the *Sox2* N1 enhancer [27,29]. The D1 enhancer had additional activity in the posterior neural plate that forms the rhombencephalon and spinal cord. The D1 sequence included CSB27, and the isolated CSB27 sequence alone displayed the same activity (see Figure 5C, below). This CSB enhancer was renamed NP1 and analyzed further. The sequence D5 enhancer showed activity in the CNS anterior to the rhombencephalon and in the lens placode, indicating a resemblance to the N3 enhancer of *Sox2*. 

### 2.3. The Time Course of the Activation of Neural Enhancers

The tissue territories of activation of the *Sox3* neural enhancers are compared in Figure 4 following the time course. Shortly after the electroporation of the tkEGFP reporter vector, the NP1 (CSB27) enhancer was activated at st.5 in the region abutting the anterior primitive streak, which is similar to the *Sox2* N1 enhancer [27,29]. Synchronous with primitive streak regression and the posterior migration of Hensen’s node, the peak position of the NP1 enhancer activity moved posteriorly. A moderate level of enhancer activity remained in the region where the enhancer was once activated, namely, in the rhombencephalon and spinal cord. Following the activation of NP1, the D13 and D15 enhancers were activated at st. 8, in the prospective diencephalon, rhombencephalon and anterior spinal cord (D13/15), and in the prospective telencephalon (D15). Later at st. 9, enhancers D4, D5 and D11 were activated in the prospective telencephalon (D4), the prospective di/mesencephalon (D5) and the spinal cord (D11). At st. 11, the CSB26 enhancer was activated in the prospective diencephalon and the medial axial levels of the spinal cord, and the D12 enhancer was activated in the ventral diencephalon. Next, at st. 15, the D6 enhancer was activated in the mes/rhombencephalon and spinal cord. These enhancers produced various overlapping patterns of their activities in the CNS primordium.

### 2.4. Functional Dissection of the NP1 Enhancer Region

In order to examine the NP1 enhancer regulation that was analogous to the *Sox2* N1 enhancer activation in axial stem cells, the DNA sequence of the NP1 enhancer was investigated in detail. A comparison of the CSB27 sequence (369 bp in the chicken) in the four vertebrate species indicated the existence of two conspicuous blocks of high sequence conservation, which were positions 52–177 (Block A) and 185–269 (Block B) (Figure 5A). 

Starting from a 1681-bp sequence, which included the 369-bp CSB27 sequence, the specific region that accounted for the enhancer sequence was narrowed down in the following steps (Figure 5B). The CSB27 sequence showed activity that was identical to the 1681-bp sequence, whereas the immediate upstream 941-bp sequence showed no activity (Figure 5B(a)). The deletion analysis shown in Figure 5B(b) indicated that the 52 to 269-bp region of the CSB27 sequence was sufficient for full enhancer activity, whereas the sequences bearing only one of the two high-conservation blocks (1–180 bp or 181–369 bp) did not show enhancer activity (Figure 5C). The 5’ step-wise deletions indicated that the 1 to 81-bp region was dispensable, but further deletion to the 92-bp position inactivated the enhancer. The 10-bp 3’ deletion from the 82 to 269-bp sequence inactivated the enhancer. Taken together, these results indicated that the 82 to 269-bp sequence was required for NP1 enhancer activity.

### 2.5. Regulation of the NP1 Enhancer by Wnt and Fgf Signals

**Figure 6:**
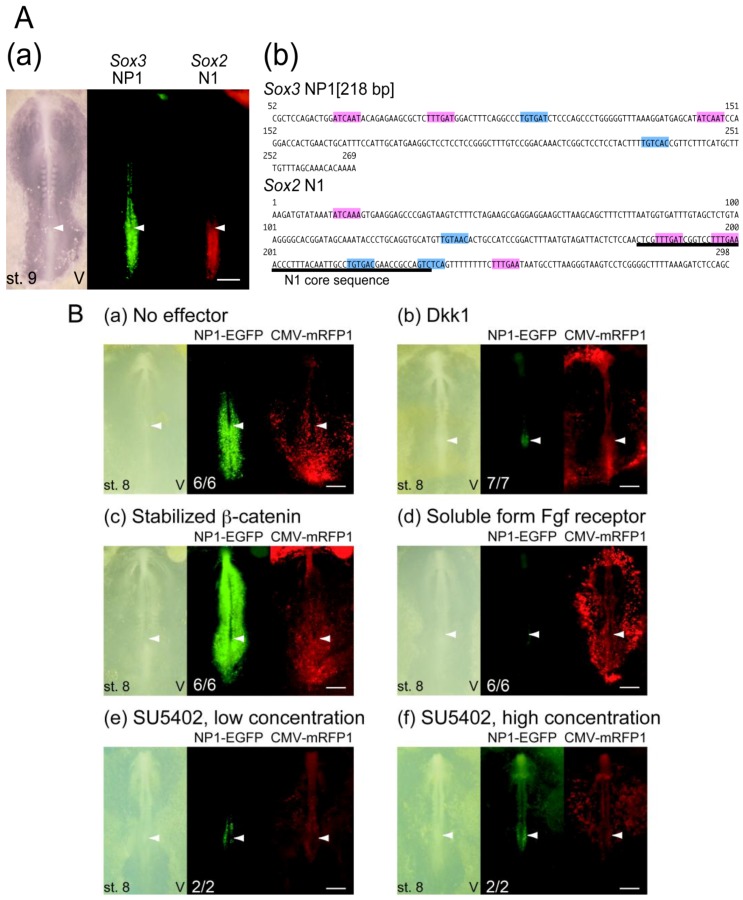
Regulation of the *Sox3* NP1 enhancer. **A.** Comparison of the *Sox3* NP1 enhancer and *Sox2* N1 enhancer. **(a)** Activities of the NP1 enhancer (NP1-tkEGFP) and N1 enhancer (N1-tkmRFP1) in the same electroporated chicken embryo at st. 9. The NP1 activity was recorded using a shorter exposure compared to other panels, in order to show the distribution of strong enhancer activity posterior to Hensen’s node. The node position is indicated by an arrowhead. **(b)** Comparison of the DNA sequences of *Sox3* NP1[218 bp] and the 298-bp *Sox2* N1 sequence [27,29]. The Lef-1 binding motifs are highlighted in pink. The Fgf-responsive element TGTGAC of N1 [29], as well as the related sequences in N1 and NP1 which are candidate elements for an Fgf response, are highlighted in blue. **B.** The dependence of NP1 enhancer activity on Wnt and Fgf signaling. Embryos were electroporated with the NP1[218 bp]-tkEGFP, pCMV/SV2-mRFP1 (electroporation control), and expression vectors for effectors modulating Wnt/Fgf signaling or treated with SU5402. After 8 hours when the embryos reached ~st. 8, the effects of the effectors on NP1 enhancer activities were assessed. The number of cases showing the same response as shown in the representative panel among the treated embryos is indicated in the NP1-EGFP panels. **(a)** A control embryo with no effectors. **(b)** Dkk1 expression that blocked canonical Wnt signaling inactivated the NP1 enhancer. **(c)** Expression of stabilized β-catenin that constitutively activated canonical Wnt signaling resulted in the strong activation of the NP1 enhancer in the entire embryonic tissue. **(d)** Expression of a soluble form of Fgf receptor that titrates out Fgf ligands inactivated the NP1 enhancer. **(e)** and **(f)** Addition of SU5402 to the culture also inactivated the NP1 enhancer. SU5402 was added at 25 μM **(e) **or 125 μM **(f)** in 50 μl yolk solution that overlaid an embryo [36], but not in the supporting agar medium. The bars indicate 500 μm.

The tissue domain that first activated the *Sox3* NP1 enhancer, namely the caudal lateral epiblast abutting the primitive streak, overlapped with that of the *Sox2* N1 enhancer as indicated in Figure 6A(a). The N1 enhancer is activated by the combined action of Wnt and Fgf signals [29,33], which is consistent with the requirement of both signals in the maintenance of the axial stem cells that activate the N1 enhancer [32,34]. Thus, whether the NP1 enhancer is also regulated by Wnt and Fgf signals was investigated. The 52-269 sequence of CSB27 contained several potential Lef1 binding sequences and putative Fgf-responsive elements (Figure 6A(b)). 

When the expression vector for Dkk1, which is an antagonist of canonical Wnt signaling, was co-electroporated with NP1-tkEGFP, the NP1 enhancer was inactivated (Figure 6B(b)). In contrast, when the expression vector for stabilized β-catenin, which constitutively activates the canonical Wnt signal pathway, was co-electroporated, the NP1 enhancer was activated in the entire embryo (Figure 6B(c)). These results demonstrated the canonical Wnt signal-dependent activation of the NP1 enhancer. Analogously, co-electroporation of a vector to express a soluble form of the Fgf receptor 1 [FGFR1c(ECD)-Fc(IgG2a)] that titrates Fgf molecules, or addition to the culture medium of SU5402, a specific inhibitor of Fgf receptor tyrosine kinase, inactivated the enhancer (Figure 6B(d)-(f)), indicating that NP1 enhancer activation also depended on Fgf signaling. These findings indicated that the *Sox3* NP1 enhancer was regulated by Wnt and Fgf signals in a manner similar to that of the *Sox2* N1 enhancer, which suggests that both *Sox2* and *Sox3* genes are regulated in an analogous manner in neural/mesodermal-bipotential axial stem cells. This would allow the regulation of these B1 Sox genes to be coordinated with the maintenance and differentiation of axial stem cells, which are also regulated by Wnt and Fgf signaling. 

## 3. Discussion

### 3.1. Regulation of the Sox3 Gene

The basal promoter of the human *Sox3* gene has been characterized as having binding sites for several tissue-nonspecific transcription factors [37,38,39], suggesting a limited contribution of the promoter in the tissue-specific regulation of the *Sox3* gene. A survey of the mouse genomic sequences within 3 kb of the *Sox3* gene with transgenic mouse embryos identified three regulatory regions that dictate transgene expression: the FB sequence of approximately 200 bp long that was located at approximately -1.2 kb in the coordinates given in Figure 1 for expression in the forebrain/midbrain /hindbrain; the V2 sequence that was located between -1.1 and 0.3 kb for expression in the V2 interneuron domain of the spinal cord; and the PNT-R sequence located between 2.8 and 4.1 kb, relative to the *Sox3* translational start site for expression in the posterior neural tube, rhombencephalon and otic vesicle [35]. Both the FB and V2 sequences were included in the C7 sequence, and PNT-R was included in the C8 sequence (Figure 1). Generation of the partial expression pattern of *Sox3* using a *X. laevis Sox3*-proximal sequence has also been reported [12].

In our present study, a region of a total of 231 kb in the chicken genome encompassing the *Sox3* locus was systematically surveyed for enhancers with the region-scanning ~5-kb DNA sequences with terminal overlaps, and 13 new enhancers that direct gene expression in specific regions of the neural and/or sensory primordia were identified. A combination of these with the mouse-determined enhancer-like elements, account for various features of *Sox3* regulation in early neuro-sensory development. However, it should be noted that our survey using chicken embryo electroporation did not detect the aforementioned *Sox3*-proximal elements that were determined using transgenic mouse embryos. One possible cause of this discrepancy was differences in the assay method. However, it probably reflected that a negatively acting element that was located around -5 kb of the *Sox3* gene masked the enhancer activities. In transgenic mouse experiments [35], it has been reported that inclusion of this region inhibited forebrain activity of the FB element. Presence of an analogous enhancer-inhibitory element has also been reported for the *X. laevis Sox3* upstream sequence [12]. The chicken DNA sequences (C7 and C8) covering the *Sox3*-proximal region were assessed as negative and included this -5 kb element, which could have masked the enhancer activities (Figure 2). 

The *Sox3* expression pattern in the chicken embryos and the activities of the newly identified enhancers are compared in Figure 3 and Figure 4. *Sox3* expression at st. 5 covered both the anterior and posterior neural plates, whereas the NP1 enhancer was active in the posterior neural plate and especially in the posterior axial stem cell region. It is likely that the FB element that was determined with the mouse system [35] regulates *Sox3* expression in the anterior neural plate. After st. 8, various enhancers that have activities in the subdomains of the forebrain-midbrain region, *i.e.*, D13, D15, D4, D5, D11, CSB26, D12 and D6, were activated in sequence. This resulted in various overlapping patterns of enhancer activities. In addition, at the rhombencephalon level, plural sequences showed enhancer activity, namely NP1, D13, D15, D6 (Figure 4) and PNT-R [35]. In the spinal cord, many enhancers showed non-uniform activity along the antero-posterior axis, with exception of the D15 enhancer. The D13 and D6 enhancers showed strong activity in the anterior half of the spinal cord at st. 11, whereas the D11 and CSB26 enhancers showed activity in the medial portion of the spinal cord (Figure 4). In the posterior region of the spinal cord, the NP1 and PNT-R enhancers exhibited activities. These observations suggested modular regulation of *Sox3* expression along the antero-posterior axis of the spinal cord that depended on the differential activities of the enhancers.

In sensory tissue development, the D5 enhancer was activated in the lens placode area at st. 11, and the CSB19 enhancer was activated at st. 12 and continued to be active until the later stages of lens development (Figure 3). Three enhancers exhibiting activity in the otic placode/vesicle have been identified: the otic placode-specific D3 enhancer, NP1 and PNT-R [35]. NP1 and PNT-R showed activity in the posterior neural plate, and the activation of these enhancers in both the otic placode/vesicle and the posterior spinal cord suggested a common signaling input in the development of the two different tissues. The U3 and D8 enhancers showed activities in the epibranchial placode regions.

### 3.2. Comparison of the Regulation of Sox3 and Sox2

We have previously shown that during the early stages of neural plate development, *Sox2* regulation is divided into non-overlapping anterior and posterior territories that are regulated by the N2 and N1 enhancers, respectively [5,27,32]. This reflects the fact that the anterior and posterior neural plates develop from the epiblast through distinct cellular mechanisms [18,32,33]. Participation of the two enhancers FB and NP1 in *Sox3* regulation in the anterior and posterior neural plate development, respectively, indicated that *Sox2* and *Sox3* are regulated on the basis of the same antero-posterior spatial division of the neural plate. 

In a detailed analysis, an important similarity was found in the regulation of the *Sox3* NP1 and *Sox2* N1 enhancers. Both of these enhancers were activated in the epiblastic tissue abutting the anterior primitive streak in which the axial stem cells reside. Both were activated by the combined action of Wnt and Fgf signals, as inhibition of either of these signals attenuated their enhancer activities (Figure 6B) [29,33]. Multiple Lef1-binding sites are found in the NP1 sequence (Figure 6A(b)), and a pair of functional Lef1-binding sites have been determined in the N1 sequence [29,33]. Putative Fgf-responsive elements were also indicated in both sequences (Figure 6A(b)). These shared features of NP1 and N1 allow for the coordinated regulation of *Sox3* and *Sox2* when the axial stem cells give rise to the neural plate and paraxial mesoderm. However, their regulation was not identical. The arrangements of the Lef1 sites and putative Fgf responsive elements were very different between NP1 and N1. In contrast to the case of the N1 enhancer, which was strongly downregulated when the neural plate was formed, the NP1 enhancer maintained its activity in the spinal cord and was also activated in the otic vesicle. 

Another analogous case had similar enhancer specificities of the *Sox3* D5 enhancer and *Sox2* N3 enhancer. These enhancers were activated in the forebrain, including the optic vesicle, and in the lens placode in st. 11 chicken embryos. It has been shown that the *Sox2* N3 enhancer is activated by the cooperative action of Sox2 and Pax6 in the diencephalon, optic vesicle and lens placode [3]. Considering the similarities in the tissue specificity of the enhancer activity, it is possible that an analogous regulation is involved in the activation of the D5 enhancer. However, their regulation was not identical, while the D5 enhancer was active in the telencephalon at st. 11, the N3 enhancer lacked activity therein. Thus, the case of the D5 enhancer represented an additional example of the analogy between the *Sox3* and *Sox2* regulatory mechanisms, yet with an appreciable difference.

Regulation of the *Sox3* gene in the neural plate progressively became more complex (Figure 4). Following the creation of the antero-posterior subdivisions by the FB and NP1 enhancers in the enhancer territories of the forming neural plate, more *Sox3* enhancers with various regional specificities were activated in sequence. The same scenario holds true for *Sox2*-regulating enhancers [27,28]. However, the activities of individual enhancers differed between *Sox3* and *Sox2* with respect to their aforementioned specificities.

The genomic arrangements of the enhancers also differed between the *Sox3* and *Sox2* genes. For instance, the D5 enhancer that was activated in the ocular primordia was located ~50 kb downstream of the *Sox3* gene, while the N3 enhancer was positioned ~15 kb upstream of the *Sox2* gene. Whereas the major *Sox3* enhancers were distributed in the region between -20 kb and 80 kb relative to the *Sox3* gene [35] (Figure 2), the *Sox2* enhancers were more widely distributed on both sides of the gene over the range of 200 kb [28]. Thus, with respect to their individual activities and genome organizations, the enhancers substantially differed between the *Sox3* and *Sox2* genes.

### 3.3. Phylogenetic Conservation of Sox3 Regulation

The wide expression of *Sox3* in the neuro-sensory primordia is common to most vertebrate species, whereas the wide expression of *Sox2* is limited to amniote animals [4]. Reflecting this fact, the individual CSBs (potential enhancers) of the *Sox3* locus were strongly conserved among the genomes of *Xenopus*, chicken and mammals (Figure 1), which is in contrast to the high degree of conservation of *Sox2*-associated CSBs that are confined to amniotes [4].

Along with these general features, species-dependent episodic changes in the enhancers were also observed. The sequence of lens-specific CSB19 enhancer of *Sox3* is conserved poorly in the mouse genome, and this may account for the absence of *Sox3* expression during mouse lens development [6]. However, the *Sox3* D3 sequence, which showed purely otic placode-specific enhancer activity (Figure 3), did not contain CSBs (Figure 2), suggesting that this enhancer was unique to avian genomes.

Conservation of genomic sequences downstream of *Sox3*, as indicated by the occurrence of CSBs common to chicken, *Xenopus* and mammals, ends beyond CSB47, which is located at approximately 90 kb in the chicken genome, whereas the conservation among mammalian sequences is extended a further 3’. Both upstream and downstream genomic regions of the *Sox3* gene appeared to be expanded in the mammalian genomes compared to the chicken and *Xenopus* genomes. An interesting issue is whether this genomic expansion is correlated with the location of *Sox3* on the X-chromosome in mammalian species. The arrangements of landmark genes in syntenic chromosomal regions indicated extensive rearrangements of the genomic domains around the *Sox3* gene in various species (Figure S1), suggesting that the genomic region investigated in this study included the major components of the *Sox3* regulatory sequences. Alternatively, the *Sox3* gene may be regulated by distant-acting regulatory sequences that withstand extensive genomic rearrangements.

## 4. Materials and methods

### 4.1. Genome Sequences of the Sox3 Locus

To analyze the enhancers and conserved sequence blocks (CSBs) in the 231-kb region spanning the *Sox3* locus in the chicken genome, we used sequence data from Gallus gallus-4.0 assembly (GCA_000002315.2) chromosome 4 genomic scaffold sequence (NW_003763735 GPS_000848988) as a reference. The central region sequence, from -23456 to 29859, covered by our bacteriophage library [27] was determined by us (DDBJ Accession number AB753847). The translational start site of *Sox3* was taken as +1 to indicate the genomic location relative to *Sox3*. This position corresponded to *Gallus gallus* chromosome 4 NW_003763735 sequence position 10474038, *Homo sapiens* chromosome X GRCh37 partial sequence position 139587000 (*Sox3* is in reverse orientation), *Mus musculus* chromosome X GRCm38 partial sequence position 60893205 (*Sox3* is in reverse orientation), *Xenopus tropicalis* genome scaffold GL172698.1 position 1000090. The CSBs were screened using VISTA Browser (http://genome.lbl.gov/vista/) using a threshold of >60% identity over a 100-bp length.

### 4.2. Enhancer Screening of the 233-kb Genomic Region and Characterization

The bacteriophage clones that carry the *Sox3*-proximal region sequences, approximately 50 kb, were used to produce the *Sox3*-proximal sequence segments C1-C13, and the two BAC clones CH261-112C13 and CH261-98D21 (ENSEMBL) were used to produce a series of ~5 kb DNA sequences with 1-kb terminal overlaps, U1 to U28 (upstream) and D1 to D17 (downstream), by PCR. These isolated sequences were individually inserted into a ptkEGFP vector [27], and electroporated at 2 μg/μL into the dorsal side of st. 4 chicken embryos. The pCMV-mRFP1 vector was also included in order to monitor the region of successful electroporation. The activation of EGFP expression at various developmental stages in New’s culture was scored as enhancer activity.

### 4.3. Effector-Dependent Regulation of the NP1 Enhancer

pNP1[218 bp]-tkEGFP at 2 μg/μL, pCMV/SV2-mRFP1 at 0.6 μg/μL and pCAGGS-based expression vectors [40] at 2 μg/μL for the expression of Dkk1, stabilized β-catenin [29] or rFGFR1(ECD)-rFc(IgG2a) (gift of Claudio Stern) were electroporated in st. 4 embryos in New’s culture. The impact of the expression of these effectors was assessed 8 hours after electroporation.

## Supplementary Materials

**Table S1:** Positioning of the conserved sequence blocks (CSBs) in the human, mouse, chicken and Xenopus genomes.

	Chicken *Sox3* locus		Human *Sox3* locus			Mouse *Sox3* locus			Xenopus *Sox3* locus		
CSB	Positions^a^	Length (bp)	Positions^a^	Length (bp)	Human *vs*. chicken Identity (%)	Positions^a^	Length (bp)	Mouse *vs*. chicken Identity (%)	Positions^a^	Length (bp)	Xenopus vs. chicken Identity (%)
1	-130257 ~ -130129	129	-970154 ~ -970026	129	84^c^	-589181 ~ -589053	129	82	Absent		
2	-110940 ~ -110700	241	-444400 ~ -444161	240	73	-522083 ~ -521837	247	74	Absent		
3	-108306 ~ -107937	370	-428513 ~ -428153	361	71	-500213 ~ -499841	373	69	-129767 ~ -129393	375	62
4	-103688 ~ -103557	132	Absent			-474215 ~ -474091	125	60	-121856 ~ -121723	134	64
5	-97501 ~ -97400	102	Absent			-388001 ~ -387900	102	62	Absent		
6	-82740 ~ -82535	206	-274707 ~ -274502	206	66	-286133 ~ -285930	204	53^d^	Absent		
7	-81558 ~ -81458	101	Absent			-297179 ~ -297065	115	60	Absent		
8	-67333 ~ -67221	113	-252300 ~ -252193	108	57^d^	-261781 ~ -261669	113	63	Absent		
9	-60650 ~ -60531	120	-246870 ~ -246750	121	64	-253372 ~ -253247	126	64	Absent		
10	-59303 ~ -59126	178	-243592 ~ -243410	183	63	Absent			Absent		
11	-56180 ~ -56079	102	Absent			-230997 ~ -230897	101	61	Absent		
12	-45556 ~ -44996	561	-156675 ~ -156165	511	75	-149792 ~ -149283	510	75	-42634 ~ -42145	490	62
13	-29736 ~ -29259	478	-137259 ~ -136775	485	79	-133617 ~ -133151	467	80	-31421 ~ -30964	458	60
14	-26388 ~ -25483	906	-88401 ~ -87521	881	78	-101763 ~ -100874	890	79	-27373 ~ -26479	895	67
15	-21081 ~ -20662	420	-34050 ~ -33637	414	77	-37248 ~ -36842	407	77	-19657 ~ -19236	422	65
16	-17784 ~ -17128	657	-28051 ~ -27389	663	82	-25441 ~ -24780	662	81	-18039 ~ -17396	644	61
17	-14950 ~ -14611	340	-23177 ~ -22843	335	76	-22166 ~ -21840	327	69	-15729 ~ -15389	341	60
18	-13560 ~ -13273	288	-22619 ~ -22337	283	65	-21624 ~ -21340	285	63	Absent		
19	-11574 ~ -11339	236	-21444 ~ -21219	226	63	-20545 ~ -20316	230	51^d^	-13988 ~ -13757	232	71
20	-5363 ~ -4690	674	-7397 ~ -6739	659	77	-6672 ~ -6014	659	76	-7241 ~ -6571	671	60
21	-4176 ~ -4004	173	Absent			Absent			-5417 ~ -5246	172	64
22	-944 ~ -792	153	-1258 ~ -1099	160	65	-1253 ~ -1097	157	65	Absent		
23	-88 ~ 997	1086	-87 ~ 1166	1254	67	-87 ~ 1177	1265	67	-88 ~ 969	1058	71
*Sox3* CDS^b^			-225 ~ 1116	1342		-225 ~ 1128	1354				
*Sox3* CDS^a^	1 ~ 951	951	1 ~ 1116	1116	66	1 ~ 1128	1128	68	1 ~ 924	924	76
24	1982 ~ 2098	117	2253 ~ 2378	126	68	2247 ~ 2369	123	64	Absent		
25	20709 ~ 20882	174	48743 ~ 48914	172	67	45466 ~ 45638	173	67	Absent		
26	24291 ~ 24916	626	54918 ~ 55541	624	70	51461 ~ 52097	637	70	22831 ~ 23470	640	70
27	29103 ~ 29471	369	64927 ~ 65286	360	63	61426 ~ 61785	360	61	24237 ~ 24595	359	76
28	33725 ~ 33839	115	Absent			Absent			28864 ~ 28991	128	63
29	35797 ~ 35915	119	114633 ~ 114750	118	61	93771 ~ 93888	118	61	31915 ~ 32036	122	61
30	37839 ~ 37943	105	Absent			Absent			50701 ~ 50801	101	69
31	43240 ~ 43490	251	146259 ~ 146505	247	60	115905 ~ 116157	253	60	Absent		
32	47105 ~ 47964	860	205211 ~ 206065	855	79	148542 ~ 149384	843	76	59011 ~ 59847	837	63
33	48341 ~ 48713	373	206395 ~ 206764	370	72	149726 ~ 150095	370	72	61798 ~ 62162	365	60
34	52194 ~ 52648	455	253981 ~ 254434	454	86	173018 ~ 173471	454	86	66646 ~ 67075	430	71
35	60282 ~ 60540	259	314971 ~ 315223	253	67	212815 ~ 213069	255	69	82061 ~ 82322	262	56^d^
36	69025 ~ 69166	142	325798 ~ 325935	138	61	235698 ~ 235833	136	62	Absent		
37	69205 ~ 69439	235	325967 ~ 326202	236	61	235872 ~ 236110	239	60	Absent		
38	71699 ~ 72240	542	346769 ~ 347310	542	82	262559 ~ 263101	543	73	93894 ~ 94430	537	65
39	75210 ~ 75377	168	376498 ~ 376664	167	84^c^	Absent			95795 ~ 95968	174	71
40	77117 ~ 77242	126	393019 ~ 393146	128	69	322436 ~ 322565	130	60	Absent		
41	77293 ~ 77414	122	393172 ~ 393292	121	62	322599 ~ 322709	111	50^d^	Absent		

^a^ The open reading frame (ORF) start site of *Sox3* is taken as 1.

^b^ Another translational start sites are annotated and conserved only in some mammalian genomes.

^c^ The case where the sequence was inverted relative to chicken sequence.

^d^ The cases where the sequence showed sequence identity vs chicken below 60%.

**Table S2:** Positioning of the DNA sequences relative to the *Sox3* ORF start site, which were tested for enhancer activity.

Sequence number	Positions relative to the *Sox3* start site (bp)	Sequence length (bp)	Enhancer activity in the CNS (HH stages^a^)	Non-CNS enhancer activity (HH stages^a^)	Analyzed specimens
U28	-134079 ~ -129036	5044	-	-	3
U27	-130087 ~ -125081	5007	-	-	7
U26	-126128 ~ -121082	5047	-	-	8
U25	-122088 ~ -117083	5006	-	-	3
U24	-118083 ~ -113121	4963	-	-	4
U23	-114084 ~ -109748	4337	-	-	4
U22	-110784 ~ -106313	4472	-	-	5
U21	-107300 ~ -102342	4959	-	-	9
U20	-103342 ~ -98324	5019	-	-	5
U19	-99343 ~ -94344	5000	-	-	6
U18	-95344 ~ -90289	5056	-	-	4
U17	-91345 ~ -86346	5000	-	-	4
U16	-87346 ~ -82309	5038	-	-	6
U15	-83347 ~ -78332	5016	-	-	8
U14	-79348 ~ -74349	5000	-	-	2
U13	-75351 ~ -70345	5007	-	-	3
U12	-71350 ~ -66311	5040	-	-	3
U11	-67351 ~ -62352	5000	-	-	4
U10	-63400 ~ -58258	5143	-	-	5
U9	-59365 ~ -54312	5054	-	-	3
U8	-55353 ~ -50146	5208	-	-	6
U7	-51354 ~ -46353	5002	-	-	9
U6	-47369 ~ -42356	5014	-	-	8
U5	-43364 ~ -38357	5008	-	-	3
U4	-39363 ~ -34454	4910	-	-	3
U3	-35550 ~ -30442	5109	-	Epibranchial placode (st. 9)	13
U2	-31484 ~ -26456	5029	-	-	3
U1	-27456 ~ -22435	5022	-	-	5
C1	-23456 ~ -16912	6545	-	-	4
C2	-22029 ~ -14489	7541	-	-	7
C3	-16915 ~ -12683	4233	-	-	4
C4	-16353 ~ -10686	5668	-	Lens placode(st. 11)	4
C5	-13647 ~ -9325	4323	-	Lens placode(st. 11)	6
C6	-10822 ~ -6096	4727	-	-	2
C7	-7397 ~ 2548	9945	-	-	5
C8	-2027 ~ 6462	8489	-	-	5
C9	4748 ~ 15637	10890	-	-	5
C10	6459 ~ 11599	5141	-	-	4
C11	13024 ~ 21441	8418	-	-	3
C12	16899 ~ 29642	12744	Diencephalon, spinal cord (st.11)	-	8
C13	21752 ~ 28244	6493	Diencephalon, spinal cord (st.11)	-	8
D1	28860 ~ 33859	5000	Posterior neural plate, rhombencephalon, spinal cord (st. 5)	Otic placode (st. 11)	15
D2	32859 ~ 37858	5000	-	-	3
D3	36858 ~ 41867	5010	-	Otic placode (st. 11)	11
D4	40830 ~ 45856	5027	Telencephalon (st. 9)	-	11
D5	44847 ~ 49855	5009	Tel/di/mesencephalon (st. 9)	Lens placode (st. 11)	12
D6	48830 ~ 53858	5029	Mes/rhombencephalon, spinal cord (st. 15)	-	8
D7	52798 ~ 57856	5059	-	-	3
D8	56853 ~ 61861	5009	-	Epibranchial placode (st. 9)	7
D9	60834 ~ 65872	5039	-	Lens (st. 17)^b^	3
D10	64835 ~ 69850	5016	Ventral diencephalon (st. 13)^b^	Otic vesicle, nasal pit (st. 13)^b^	3
D11	68843 ~ 73849	5007	Spinal cord (st. 9)	-	10
D12	72849 ~ 77848	5000	Diencephalon (st. 11)	-	11
D13	76848 ~ 81847	5000	Posterior neural plate, di/rhombencephalon, spinal cord (st. 8)	-	12
D14	80847 ~ 85895	5049	-	-	3
D15	84850 ~ 88760	3911	CNS (st. 8)	-	3
D16	88029 ~ 93228	5200	-	-	3
D17	92231 ~ 97230	5000	-	-	5
CSB19	-12377 ~ -10964	1414	-	Lens placode (st. 11)	3
CSB26	24264 ~ 25312	1049	Diencephalon, spinal cord (st. 11)	-	5
CSB27	28179 ~ 29859	1681	Posterior neural plate, rhombencephalon, spinal cord (st. 5)	Otic placode (st. 11)	37

^a^ The earliest developmental stages recorded for the EGFP expression are indicated.

^b^ These showed the low enhancer activities, and data were not shown in this text.
